# A Review of Simulation Tools for Thin-Film Solar Cells

**DOI:** 10.3390/ma17215213

**Published:** 2024-10-25

**Authors:** Lizbeth Salgado-Conrado, Carlos Álvarez-Macías, Bernardo Reyes-Durán

**Affiliations:** 1 Facultad de Ingeniería Mecánica y Eléctrica, Universidad Autónoma de Coahuila, Carr. Torreón-Matamoros, km 7.5, Torreón 27276, Coahuila, Mexico; 2 Tecnológico Nacional de México/Instituto Tecnológico de La Laguna, Torreón 27000, Coahuila, Mexico; d.breyesd@correo.itlalaguna.edu.mx

**Keywords:** thin-film solar cells, numerical simulation tools, photovoltaic performance, simulation software comparison, material modeling

## Abstract

Unlike current silicon-based photovoltaic technology, the development of last-generation thin-film solar cells has been marked by groundbreaking advancements in new materials and novel structures to increase performance and lower costs. However, physically building each new proposal to evaluate the device’s efficiency can involve unnecessary effort and time. Numerical simulation tools provide a solution by allowing researchers to predict and optimize solar cell performance without physical testing. This paper reviews thirteen of the main numerical simulation tools for thin-film solar cells, including SCAPS, AMPS, AFORS-HET, ASPIN3, GPVDM, SESAME, SILVACO, SENTAURUS, and ADEPT. This review evaluates each tool’s features, modeling methods, numerical approaches, and application contexts. The findings reveal notable differences in material modeling, numerical accuracy, cost, and accessibility among the tools. Each tool’s strengths and limitations in simulating thin-film solar cells are highlighted. This study emphasizes the necessity of selecting suitable simulation tools based on specific research requirements. It provides a comparative analysis to assist researchers in choosing the most effective software for optimizing thin-film solar cells, contributing to advancements in photovoltaic technology.

## 1. Introduction

The development of solar cells has evolved through various generations, with traditional thick crystalline silicon wafers leading to second-generation thin-film solar cells, which have reduced costs by using less material and expanding production areas [1]. Solar cell performance is closely linked to the materials used, and as manufacturing costs decrease, the focus shifts to material costs and fabrication techniques [2,3]. Simulation tools have become essential for analyzing and optimizing solar cell designs, avoiding the high costs and time associated with physical manufacturing [4].

Early reviews, such as Bugelman et al. [5], provided overviews of software like ASA, SCAPS, and PC1D, emphasizing the importance of comparing simulations with experimental results. Abou-Ras et al. [6] and Zhang and Yeon-Gil [7] reviewed tools like AFORS-HET, AMPS-1D, ASA, and SCAPS, discussing their features and accuracy. Haddout et al. [8] highlighted the role of modeling in understanding CZTS-based solar cells, while [9] offered a comparison of solar cell simulators.

This paper addresses the lack of comprehensive research on simulators for thin-film solar cells by reviewing thirteen tools, including SCAPS, AMPS, AFORS-HET, ASPIN3, GPVDM, SESAME, SILVACO, SENTAURUS, and ADEPT. We evaluate their features, advantages, and limitations, comparing them across materials, modeling methods, cost, and accuracy to provide insights into their effectiveness in advancing solar cell research.

## 2. Methodology

The methodology for evaluating software tools used in the simulation and modeling of solar cells involved several vital steps. First, a comprehensive literature review was conducted to identify and select relevant software based on their capabilities and usage in academic research. Criteria such as the ability to model electrical, optical, and thermal phenomena, ease of use, cost, and advanced features were considered. Standard thin-film solar cell structures were set up in each software with consistent simulation parameters to ensure uniformity. Numerical methods employed by each tool were examined for their effectiveness and efficiency. A comparative analysis was performed to evaluate the accuracy, computational efficiency, and cost of each software, with validation against experimental data ensuring the reliability of results. Finally, the findings were documented in a structured format, highlighting each tool’s insights, strengths, and limitations.

## 3. General Aspects of Numerical Simulation

Numerical simulation for thin-film solar cells involves various materials, numerical methods, and critical simulation parameters. Simulators can model a range of semiconductors, including CdTe, CIGS, amorphous silicon, and kesterite compounds, each with distinct properties that impact their photovoltaic performance. Advanced numerical methods, such as differential equation solvers and finite element methods, are employed to accurately represent phenomena like light absorption, carrier transport, and recombination. Critical simulation parameters, including temperature, incident illumination, and electrical contact conditions, are fine-tuned to align with experimental conditions, ensuring the model’s accuracy and optimization. This approach allows researchers to explore designs and operational scenarios, providing valuable insights for advancing thin-film photovoltaic technologies. This section briefly overviews these simulations’ most commonly used materials, methods, and parameters.

### 3.1. Types of Photovoltaic Cells and Materials

Different semiconductor materials and technologies were introduced for designing cost-effective and high-efficiency solar cells. According to Martin Green’s classification [10], the first generation includes silicon wafer-based technology. The second generation comprises thin-film technologies, which use inorganic materials and feature absorbent layers that are a few micrometers thick, typically single junctions. The third generation encompasses thin-film solar cells, including emerging technologies such as perovskite, multi-junction cells, quantum dots, intermediate band gaps, and hot carrier cells. A good example is the popular perovskite cells operating differently than conventional p–n junction cells. Hybrid organic–inorganic perovskite (HOIP) photovoltaics have emerged as a promising new technology, owing to their rapidly increasing efficiency. To further enhance the benefits, a perovskite–silicon tandem device was proposed to commercialize perovskite photovoltaics, leading to higher power conversion efficiency [11]. These technologies aim to surpass the Shockley–Queisser limit through nanostructured, organic, inorganic, and hybrid materials [12]. Emerging photovoltaic technologies also potentially address future challenges by integrating with other technologies to create intelligent, compact systems that efficiently harness collected energy.

Simulation analysis is a critical tool in developing solar cells of these three generations, including technologies based on emerging materials (whether single-junction or tandem cells), utilizing platforms SILVACO, SCAPS, COMSOL, and wxAMPS [13]. These tools identify design issues and propose potential solutions, as the simulation phase predicts the cells’ performance before fabrication, thereby saving time and costs. Most of these simulation programs are based on the Shockley–Queisser limit, which describes the maximum achievable solar energy conversion efficiency for specific materials of a single junction solar cell, and the majority does not consider emerging third-generation technologies. Therefore, these programs may be strongly inaccurate if they do not consider specific quantum corrections [14,15]. However, some of them are being adapted to emerging photovoltaic technologies, which offer an essential route to higher-efficiency photovoltaic devices. For instance, Faiza Azri used the SCAPS simulator to study the basic structure of perovskite-based solar cells and improved their performance by optimizing the electron and hole transport layers (ETL and HTL, respectively) [16].

Table 1 provides an overview of the material options categorized by generation type currently used for developing and simulating solar cells [17].

### 3.2. Types of Modeling Used in the Simulation

In the simulation, various modeling techniques are employed to accurately represent complex systems and predict their behavior under different scenarios. These models range from deterministic approaches, which provide precise outcomes based on fixed inputs, to stochastic models that account for randomness and uncertainty.

In this section, we will present the most commonly used models in the simulation of thin-film solar cells and provide a brief description of each [6]:

1. Electronic and Optical Properties Modeling
  (a) Band Diagram Modeling: visualization of the energy band structure, including conduction and valence bands, Fermi levels, and band-bending effects.
  (b) Quantum Efficiency (QE) Modeling: calculation of the external and internal quantum efficiency helps us understand the wavelength-dependent response of the solar cell.
  (c) Spectral Response Modeling: evaluation of the spectral response to determine how different wavelengths of light affect the photocurrent generation.
  (d) Optical Modeling: incorporation of optical properties, such as absorption, reflection, and transmission of light within the solar cell structure, is essential for designing anti-reflective coatings.
  (e) Light Trapping and Scattering Modeling: incorporation of light trapping and scattering mechanisms enhances absorption in thin-film solar cells.
2. Electrical and Transport Phenomena Modeling
  (a) Current–Voltage (I–V) Modeling: this technique involves analyzing the current–voltage characteristics under various illumination and temperature conditions, which is crucial for evaluating cell efficiency and performance.
  (b) Electrical Modeling: simulates the electronic behavior of solar cells, including charge transport, generation, and recombination.
  (c) Carrier Transport Modeling: simulating carrier transport mechanisms, including Drift–Diffusion equations for electrons and holes, allows for the analysis of recombination and generation rates.
  (d) Recombination Mechanism Modeling: detailed analysis of recombination mechanisms, including Shockley–Read–Hall, Auger, and radiative recombination, to understand loss mechanisms and improve efficiency.
  (e) Series and Shunt Resistance Modeling: analysis of the impact of series and shunt resistances on the I–V characteristics and overall efficiency.
  (f) Material Properties Modeling: simulation of the influence of different material properties, such as bandgap, mobility, and permittivity, on the performance and efficiency of the solar cell.
  (g) Capacitance Modeling: simulation of the capacitance–voltage characteristics provides insights into the charge storage and dielectric properties of the solar cell layers.
  (h) Electron Transport Layer (ETL) and Hole Transport Layer (HTL) modeling: ETLs and HTLs are pivotal in charge transport, separation, and recombination [11]. Their thickness, carrier concentration, and associated bulk defects must be adjusted to obtain the best cell performance with superior stability [37].
3. Device Structure and Interface Modeling
  (a) Doping and Defect Modeling: simulation of the effects of doping concentrations and defect states on the solar cell’s electronic properties and overall performance.
  (b) Interface Modeling: examination of the properties and effects of interfaces between different layers in the solar cell, crucial for multi-junction and heterojunction cells.
  (c) Multi-Junction Modeling: simulates tandem and multi-junction solar cells, accounting for the interaction between different sub-cells.
4. Thermal and Transient Response Modeling
  (a) Thermal Modeling: analyzes the thermal effects within solar cells, accounting for heat generation and dissipation.
  (b) Transient Response Modeling: modeling of the solar cell’s transient response to changes in illumination or bias conditions, useful for dynamic performance analysis.
5. Performance Metric Modeling
  (a) Photocurrent and Photovoltage Modeling: analysis of the generation and collection of photocurrent and the development of photovoltage under various illumination conditions.
  (b) Lifetime and Degradation Modeling: this technique involves analyzing solar cells’ long-term performance and degradation over time under various environmental and operational conditions.
6. Multiscale and Noise Modeling
  (a) Multiscale Modeling: this technique combines models at different scales, from quantum mechanical to macroscopic, to capture the full range of phenomena in solar cells.
  (b) Stress Effects: this simulation simulates the impact of mechanical stress on solar cell performance, which is relevant for understanding reliability and durability under varying conditions.
  (c) Noise Modeling: This technique analyzes noise characteristics within solar cells, providing insights into device performance in noisy environments or under varying operational conditions.

Table 2 summarizes the strengths, weaknesses, and uses of different types of modeling employed in thin-film solar cell research.

### 3.3. Numerical Methods Used in the Simulation

Numerical methods play a pivotal role in the simulation of thin-film solar cells, providing the tools necessary to solve complex equations that describe the physical processes within these devices. These methods enable researchers to model charge transport, light absorption, and recombination dynamics with high precision, leading to a deeper understanding of the factors that influence solar cell performance [6]. This section will explore the key numerical techniques employed in the simulation of thin-film solar cells, highlighting their application [46].

1. Numerical Methods for Differential Equations
  (a) Finite Element Method (FEM): this method models physical behavior like heat flow and charge transfer by discretizing the device structure into finite elements. It is widely used for complex simulations.
  (b) Finite Difference Method: this method discretizes continuous domains into a mesh of points to approximate spatial derivatives. It is useful for solving diffusion and recombination equations.
  (c) Finite Volume Method (FVM): this method analyzes heat transfer and fluid dynamics by integrating over discrete volumes. It handles complex geometries and optimizes performance.
  (d) Euler Method: this method is used in solar cell software for temporal discretization, energy generation calculations, and parameter identification. Its accuracy and stability depend on the specific application and time step choice.
  (e) Drift–Diffusion Modeling: this method simulates solar cells using the steady-state Drift–Diffusion model, which is a fundamental model for semiconductor device
2. Matrix and Iterative Methods
  (a) Transfer Matrix Method (TMN): this method calculates optical properties and light interaction with solar cell materials, enhancing design efficiency.
  (b) S-Matrix Method: this method models the optical properties of solar cells, including absorption profiles and electric field distributions, which are crucial for understanding charge carrier generation and transport.
  (c) Gummel’s Method: a decoupled approach to solving Drift–Diffusion and Poisson’s equations iteratively, improving stability and convergence.
  (d) Newton–Raphson Method: solves nonlinear algebraic equations resulting from discretization, refining solutions iteratively.
3. Statistical and Quantum Mechanics Methods
  (a) Fermi–Dirac Statistics: Fermi–Dirac statistics are vital for modeling solar cells, particularly with high doping. Tools like PC1D use these statistics to improve simulation accuracy and optimize silicon solar cell performance.
  (b) Monte Carlo Method: allows us to analyze the behavior of light and charge transport within these devices. SCAPS is a tool that uses this method to model complex processes.
4. Advanced Structures and Materials Mode
  (a) Multi-Quantum Well Structures (MQW): Combining optical and electrical modeling techniques enhances light absorption and efficiency.

The Finite Element Method (FEM) and Finite Difference Method (FDM) are both foundational techniques for solving partial differential equations, though they differ significantly in their applications and computational demands. The FEM excels in handling complex geometries and is widely used in multi-physic simulations, providing higher-order accuracy at the cost of more excellent computational resources [47]. While simpler and more computationally efficient, the FDM is less suited to problems involving irregular geometries and tends to produce less accurate results [48]. The Finite Volume Method (FVM), commonly employed in computational fluid dynamics, balances computational efficiency and accuracy by averaging values over control volumes [49]. Specialized techniques, such as Gummel’s Method, are essential for solving nonlinear semiconductor equations, while the Newton–Raphson Method remains a reliable choice for ensuring convergence in nonlinear systems [50]. Euler’s Method, although straightforward, is often inadequate for complex, stiff equations due to its limited stability [51]. A summary of the strengths and applications of various numerical methods commonly used in the simulation and analysis of thin-film solar cells is presented in Table 3.

## 4. Brief Description of Computational Tools

### 4.1. SCAPS

**Figure 1:**
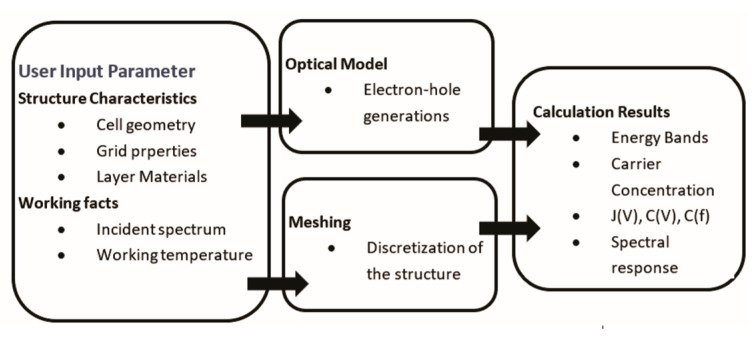
Basic diagram of SCAPS work operation [62].

Initially designed for CdTe and CIGS solar cells and developed at the University of Gent, Belgium, SCAPS now supports various cell types, including Si, GaAs, and a-Si [61,62]. SCAPS models optical and electrical properties, visualizes energy band structures, and can handle up to seven semiconductor layers with diverse materials and doping profiles [62]. It uses drift–diffusion equations for carrier transport, incorporates light trapping and scattering models, and simulates I–V characteristics, quantum efficiency, spectral response, and fabrication processes [62] (Figure 1).

SCAPS has limitations, such as simplified optical models that may affect accuracy, especially for complex or multi-layered structures [63]. It can struggle with intricate material compositions and interfaces and may have longer simulation times or inefficiencies with complex structures [62,63]. Despite these challenges, SCAPS is valuable for its customizable configurations, access to internal variables, and calibration with experimental results.

### 4.2. AMPS

**Figure 2:**
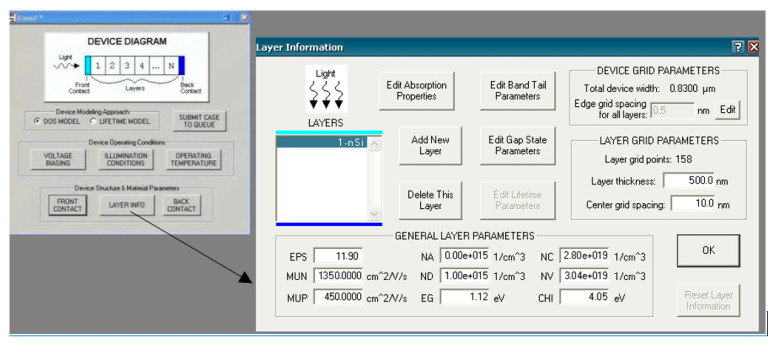
Selecting the layers and parameters in AMPS-1D [68].

The Analysis of Microelectronic and Photonic Structures (AMPS) is a one-dimensional simulator designed for CIGS solar cells, including homojunction, heterojunction, and multi-junction structures. It supports crystalline, polycrystalline, and amorphous materials. It simulates thin-film solar cells under various conditions, such as light intensity, temperature, and material properties, while calculating parameters like electrostatic potential and carrier current densities [64,65,66,67,68]. The AMPS can also simulate thin-film deposition processes like CVD and PVD to predict film properties [68]. The software’s three-stage simulation process covers the operational environment, material properties, and results [66,67]. AMPS’s strengths include modeling deposition processes and offering interactive tools for analyzing performance metrics. Its user-friendly GUI is shown in Figure 2, and examples further enhance the modeling precision for cells with high defect densities [67].

However, the AMPS has limitations due to its one-dimensional nature. It struggles with simulating solar cell structures that require two-dimensional or three-dimensional simulations, such as complex multi-junction cells with intricate interfaces. Additionally, its reliance on atomistic modeling demands significant computational resources and time, especially for large-scale systems or lengthy simulations. These constraints make it less suitable for scenarios involving complex geometries or varying environmental conditions that influence solar cell performance [69].

### 4.3. ASA

The Advanced Semiconductor Analysis (ASA) is a one-dimensional simulator from Delft University of Technology, ideal for multi-layer heterojunction semiconductors, including both amorphous and crystalline devices [70]. It optimizes c-Si wafer and tandem solar cells by integrating optical and electrical models to accurately predict I–V curves, fill factors, and efficiencies [71]. The Genpro4 optical model in the ASA enhances calculations for complex structures, making it valuable for indoor light harvesting in applications like building-integrated photovoltaics [72]. While the ASA offers flexibility with script-based inputs and external programming support, its one-dimensional nature may limit simulations of complex geometries and diverse environmental conditions. It also requires a strong understanding of semiconductor physics, which may be challenging for beginners [73].

### 4.4. AFORS-HET

**Figure 3:**
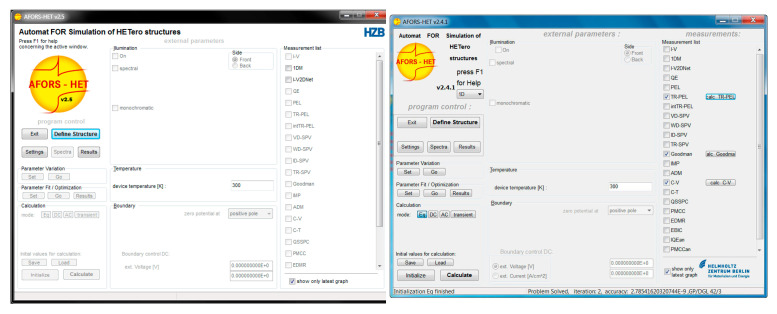
Main windows of AFORSHET.

The AFORS-HET (Automat for Simulation of Heterostructures) is a one-dimensional simulation tool that Helmholtz-Zentrum Berlin developed explicitly for analyzing heterojunction solar cells. It is particularly effective for modeling devices that combine amorphous and crystalline materials, such as silicon heterojunction solar cells [74]. The software excels in simulating solar cells’ optical and electronic properties, enabling a detailed analysis of band alignment, charge carrier transport, and recombination mechanisms [75]. Its user-friendly interface makes it accessible for researchers and engineers, as shown in Figure 3.

However, AFORS-HET’s one-dimensional approach may not fully capture the complexities of three-dimensional structures or intricate geometries, potentially limiting its accuracy for solar cells with multiple interfaces or complex interactions. Additionally, while it performs well under standard test conditions, the AFORS-HET may struggle to accurately model the effects of diverse environmental conditions, such as temperature variations and non-uniform illumination, which is critical for real-world solar cell performance [76].

### 4.5. SC-SIMUL

SC-SIMUL is a software tool for simulating solar cells, focusing on amorphous silicon-crystalline silicon heterojunctions and amorphous semiconductors. It models light absorption, reflection, and transmission, considering material composition, thickness, and surface morphology [77]. The software features an intuitive interface for 2D and 3D visualization, data analysis, and report generation, making it accessible to researchers and engineers [78]. However, as a primarily one-dimensional simulator, SC-SIMUL may struggle with complex 3D structures and real-world environmental variables, potentially limiting its accuracy and applicability [79].

### 4.6. ASPIN3

The ASPIN3 is a two-dimensional semiconductor device simulator based on the steady-state drift–diffusion model for simulating diodes, transistors, and solar cells [80]. It optimizes device design by modeling carrier behavior and can be integrated with optical simulators like SunShine for solar cell analysis [81]. ASPIN3 accurately models electrical, optical, and thermal processes with a user-friendly interface for result visualization [82,83]. However, it demands significant computational power for complex simulations and may struggle with non-standard environmental conditions, limited to rectangular structures [80,81,82]. Additionally, it has a steep learning curve for new users.

### 4.7. GVPDM

The GPVDM (General Purpose Photovoltaic Device Model) is a versatile software tool for simulating various solar cells, including thin-film, organic, and perovskite devices. It integrates optical and electrical modeling, providing detailed insights into device behavior. Its user-friendly interface and robust visualization tools make it accessible to users of different expertise levels, supporting 1D and 2D simulations across various configurations [84,85,86,87,88].

However, the GPVDM’s detailed physical models can lead to high computational demands, particularly for large-scale or intricate simulations. Features like a 3D thermal solver and exciton diffusion model add to the complexity. While compelling under standard conditions, the GPVDM may struggle with varying environmental factors such as temperature changes and light intensities, especially for silicon and thin-film solar cells. It also assumes defect-free perovskite layers, which may not accurately reflect real-world scenarios [86,87,88]. Additionally, new users may face a steep learning curve when fully utilizing its advanced features.

### 4.8. SESAME

**Figure 4:**
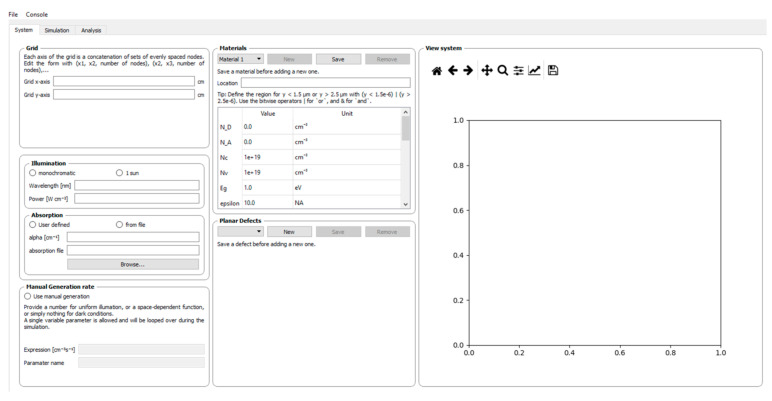
Sesame software simulation.

SESAME is an open-source Python package for simulating polycrystalline photovoltaics, including grain boundaries and surfaces. It supports 1-D and 2-D systems like CdTe, CIGS, and hybrid perovskites [53,89]. It models electrical, thermal, and optical properties, providing detailed predictions of metrics such as efficiency and voltage. SESAME offers rapid parameter exploration and visualization tools for charge transport and defects, with a user-friendly interface [90,91], as shown in Figure 4.

Due to its detailed physical modeling, SESAME faces limitations, leading to high computational demands, especially for large or complex simulations. It solves 1D and 2D Drift–Diffusion–Poisson equations, which are computationally intensive [53]. The software is limited to non-degenerate semiconductors using Boltzmann statistics and does not support thermionic emission or quantum tunneling [91]. Its ability to handle 3D systems is untested, which may restrict its use for more complex structures [89]. Additionally, SESAME may struggle with non-standard environmental conditions, impacting real-world predictive accuracy, and it has a steep learning curve for new users [90].

### 4.9. SILVACO

SILVACO provides advanced 2D and 3D simulation software for semiconductor devices, modeling thin-film materials like a-Si, CIGS, and CdTe, including multi-junction and heterojunction cells [92]. It offers a detailed electrical and optical property analysis, using techniques such as trap-limited carrier transport and solving transport, Poisson, and diffusion equations [92,93,94]. The software features user-friendly interfaces for parameter extraction, optimization, and data visualization, with ATENA and ATLAS models for in-depth analysis [95,96,97].

Limitations include potential challenges in accurately modeling perovskite solar cells and complex geometries, such as interdigitated back-contact cells [93]. The software’s high computational demands can be taxing, and its complexity may require extensive training [96]. Additionally, SILVACO lacks features for simulating thermionic emission and quantum tunneling, which could impact the accuracy of specific heterojunction devices [97].

### 4.10. PC1D

**Figure 5:**
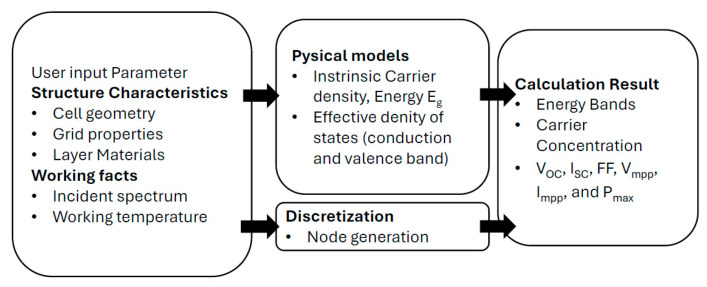
Basic diagram of PC1D work operation [100].

The PC1D (Personal Computer One Dimensional) is an open-source program developed for computing the performance of various photovoltaic structures, including GaAs, a-Si, Al-GaAs, Si, InP, and Ge [98]. This powerful simulation tool allows researchers to simulate factors such as the impact of band gap and electron affinity tuning for improved performance. The PC1D operates by discretizing the structure to be simulated, focusing on nodes in regions with doping changes or near surfaces [99], as shown in Figure 5. To enhance accuracy, the software incorporates advanced models like trap-assisted tunneling, intra-band effects, and Fermi–Dirac statistics. It can model recombination mechanisms such as Auger, band-to-band, and trap-assisted tunneling [100].

The PC1D offers a user-friendly interface and straightforward setup, making it suitable for educational purposes and early-stage research [101]. Its ability to extract device parameters and compare simulated results with experimental data is valuable for optimizing performance.

However, PC1D’s one-dimensional nature limits its capacity to model complex designs like multi-junction cells or those with intricate geometries, potentially affecting accuracy in J_sc_ and V_oc_ [102]. It may also struggle with shading effects and intricate environmental conditions, and it cannot define a general Density of States (DOS) distribution, limiting its use for materials where deep state charge is significant, such as II-VI or a-Si solar cells [98]. Despite these limitations, it remains useful for basic simulations and educational purposes.

### 4.11. Sentaurus TCAD

Sentaurus TCAD is a multidimensional software for modeling and optimizing semiconductor devices, including thin-film solar cells made from materials like a-Si, CdTe, and CIGS, across one, two, and three dimensions [103]. It uses advanced modeling techniques to simulate electrical and optical properties, carrier transport, recombination, and conversion efficiency under various conditions [104]. The software is scalable and capable of simulating individual devices to entire modules. It supports many devices, including MOSFETs, FinFETs, and solar cells, within a robust GUI-driven environment [103]. Sentaurus TCAD also offers flexible meshing strategies to balance accuracy and simulation time.

However, it has limitations, particularly in modeling solar cells with complex geometries and lateral variations, such as interdigitated designs and grain boundaries [103]. TCAD simulations are computationally intensive, requiring high-performance computing resources, and can face challenges in convergence and stability, especially with high-field and avalanche generation models [105]. Despite these challenges, Sentaurus TCAD is a powerful tool that provides valuable insights for optimizing thin-film solar cells and other semiconductor devices, helping to reduce the need for physical prototypes and saving time and resources in development [106].

### 4.12. ADEPT

ADEPT (A Device Emulation Program and Toolbox) is a versatile numerical simulator for modeling solar cells across one, two, and three spatial dimensions, supporting configurations such as single, thin-film, and multi-junction solar cells [107,108]. It solves Poisson’s and continuity equations using the generalized Newton method, enabling analyses like I–V characteristics, spectral response, and capacitance–voltage profiles [107]. ADEPT is highly customizable, allowing users to modify parameters for new device structures and adapt to various geometries. However, simulations in 2D and 3D require significant computational resources. While ADEPT is accessible online and user-friendly, it may have limitations compared to more comprehensive TCAD tools, offering a narrower range of supported materials and device architectures. Experimental validation is advised for novel designs to ensure accuracy [108].

### 4.13. QUOKKA

QUOKKA, developed by Andreas Fell, is a specialized software for simulating and optimizing solar cells, mainly focusing on 1D, 2D, and 3D charge carrier transport in configurations like interdigitated back contacts (IBC) and front and rear contacts (FRC) within quasi-neutral silicon structures [109]. It offers a comprehensive database of materials, such as a-Si, CdTe, and CIGS, and supports simulations under various environmental conditions to optimize solar cell designs [110]. The software uses simplified semiconductor carrier transport models, balancing computational efficiency with accuracy, and supports the detailed analysis of steady-state electrical characteristics [111]. QUOKKA’s user-friendly interface and visualization tools make it accessible for researchers and industry professionals and scalable for different applications [112].

However, QUOKKA has a steep learning curve due to its sophisticated modeling and requires detailed input data for accurate predictions, especially for novel structures. It may struggle with non-rectangular device shapes or surface morphologies, and its conductive boundary simplifications can limit the detailed modeling of near-surface regions, such as emitter diffusion [110,111]. The software is designed for typical solar conditions and may perform poorly under exotic or extreme conditions. Additionally, QUOKKA cannot simulate reverse breakdown effects, and its meshing approach restricts it to cuboidal solution domains, posing challenges for non-rectangular geometries [113].

## 5. Comparison of Computational Tools for Thin-Film Solar Cells

The numerical tools used in solar cell simulation were compared based on their ability to model different materials, handle various parameters, employ specific numerical methods, and achieve accurate results. Each tool was assessed for its computational efficiency and scalability, which are key factors in thin-film photovoltaic research. A comparative table showcases thirteen solar simulator software packages, highlighting their compatibility with different generations of solar cell materials. These range from first-generation materials like silicon (Si) and gallium arsenide (GaAs) to advanced third-generation materials such as perovskites and multi-junction cells. This comparison underscores each tool’s versatility and applicability across various domains of photovoltaic research.

### 5.1. Photovoltaic Cells and Materials

Developing new solar cell technology involves adjusting various parameters, where factors such as material type, geometric arrangement, and thickness may be modified to improve and enhance device performance. Computational modeling simulators have made it easier and more accessible to evaluate these parameters, eliminating the need to physically build and test every new change. However, the lack of quantum corrections in the conventional numerical modeling of solar cells employed by most commercial packets causes a giant inaccuracy in nanoscale materials. Given the limited information available and the variety of software tools, selecting the appropriate software tool for developing a thin-film solar cell can be challenging.

For the device model to be reliable, the software must be equipped with accurate information about the chosen material, including layer measurements and technology, the architecture of the desired structure, physical sensing, and sensitivity. For instance, PC1D is typically used for crystalline silicon solar cells, whereas SCAPS-1D is employed for CIGS and, more recently, perovskite. An ASA is primarily for amorphous silicon, a GPVDM is utilized for organic solar cells, AFORS-HET is designed for heterojunction solar cells, and AMPS-1D, SCAPS-1D, and ADEPT are used for multiple solar cell simulations. Most simulation software is based on single-junction solar cell models, although the ASA and ADEPT offer additional features for simulating lower-efficiency tandem solar cells. In contrast, wxAMPS can simulate III-V multi-junction solar cells, surpassing typical single-junction solar cell simulators. Table 4 presents the main generations of solar cell materials used by these 13 software programs to simulate different layers in the design of a thin-film solar cell.

### 5.2. Modeling Used in the Simulation

All the programs studied in this paper are powerful tools for modeling various characteristics and behaviors of photovoltaic devices. They simulate aspects such as band diagrams, capacitance, current–voltage (I–V) curves, quantum efficiency, carrier transport, optical effects, doping and defect modeling, transient response, spectral response, series and parallel resistance, and hole/electron transport layer and interface modeling (see Table 5). These capabilities enable users to investigate and optimize solar cell designs, understand operational mechanisms, and predict performance under different conditions. The versatility of these simulation tools is essential for advancing and enhancing photovoltaic technology.

As Table 5 highlights, several programs excel in specific areas like degradation modeling, lifetime, multi-junction, multiscale, stress effects, and noise. For example, GPVDM can simulate the effects of degradation mechanisms on solar cell performance, modeling the impacts of bias, photo-stress, and wear on devices like organic solar cells, OLEDs, and OFETs [5,114]. In lifetime modeling, tools like SCAPS, AMPS, AFORS-HET, ASPIN3, GPVDM, SESAME, SILVACO, SENTAURUS, and ADEPT allow users to input lifetime values into the Shockley–Read–Hall recombination model, offering a simplified view of the device performance [115,116,117,118,119,120,121]. For multi-junction modeling, programs such as SCAPS, AMPS, AFORS-HET, SESAME, SILVACO, PC1D, SENTAURUS, and ADEPT can simulate multi-junction solar cells across 1D, 2D, or 3D, making them versatile for various tandem structures like perovskite/silicon and CIGS/c-Si [117,119,122]. However, for stress effects, tools like AFORS-HET, SC-SIMULC, SESAME, and SENTAURUS lack sufficient details on their modeling approaches [123,124]. Lastly, SCAPS is noted for noise modeling, simulating thermal noise, shot noise, and 1/f noise, which are critical for understanding defects and traps in semiconductors [125,126,127,128,129].

### 5.3. Analysis of Numerical Methods Used in the Simulation

As shown in Table 6, the programs used for simulating and optimizing thin-film solar cells employ various numerical methods to evaluate their performance, which include the following: 1. numerical methods for differential equations allow modeling the complex physics of solar cells, including charge transport and optical effects at the device level; 2. matrix and interactive methods analyze multilayer solar cells’ spectral response and quantum yield; 3. statistical and quantum mechanics are used for charge transport analysis in solar cells with complex materials; and 4. advanced structure and material mode include studying novel materials such as perovskites and multi-junction solar cell structures [130,131,132,133,134,135,136].

In the literature, most software packages utilize the Finite Element Method (FEM) in various ways. SCAPS uses the FEM to simulate the electrical and optical behavior of solar cells, focusing on potential distribution and charge carrier generation [127,128,129,130,131,132,133]. AMPS applies the FEM for thermal and electrical analysis of thin-film solar cells, considering temperature effects and operating conditions [132]. ASA allows for component-level simulation, offering detailed behavior analysis under different conditions [133,134]. AFORS-HET uses FEM to simulate heterostructures, analyzing material interactions and performance [75,117]. SC-SIMUL focuses on current and voltage distribution in solar cell simulations [124]. ASPIN3 employs FEM for designing optoelectronic devices like LEDs and lasers, emphasizing precision in electromagnetic field simulations [80,135,136,137]. GPVDM applies FEM to photovoltaic device simulations, analyzing charge carrier generation and recombination [84,86]. SESAME uses FEM for simulating semiconductor devices and electronic systems, focusing on charge distribution and electric fields [89,90]. SILVACO applies FEM across a broad range of electronic and semiconductor devices, including transistors [138,139,140]. PC1D and QUOKKA use FEM for detailed analysis of photovoltaic device efficiency and performance [140,141,142]. SENTAURUS employs FEM for advanced semiconductor device simulations, including transistors and sensors [143,144]. ADEPT uses FEM to simulate electronic and semiconductor devices, providing detailed analysis of electronic and optical properties [138,145].

The Finite Difference Method (FDM) is widely used in various simulation programs to analyze semiconductor devices and solar cells. SCAPS employs the FDM to solve Poisson and continuity equations, simulating the electrical characteristics of thin-film solar cells [145,146,147]. The AMPS uses the FDM to calculate potential distribution, carrier concentration, and electric fields in semiconductor devices [148,149]. The ASA applies FDM for component-level simulations, offering insights into device behavior under different conditions [150]. AFORS-HET utilizes the FDM to model heterojunctions, examining material interactions on device performance [151,152,153,154,155,156]. SC-SIMUL focuses on analyzing solar cells’ current and voltage distribution [78]. ASPIN3 uses the FDM for optoelectronic device design, including LEDs and lasers, focusing on electromagnetic field simulations [82]. The GPVDM applies the FDM for detailed photovoltaic device analysis, including electrical and optical characteristics [155]. SESAME focuses on simulating charge and electric field distribution in semiconductor devices [89]. SILVACO uses FDM for various electronic and semiconductor devices, including transistors [156,157,158]. PC1D leverages FDM for analyzing photovoltaic device efficiency [99,101]. SENTAURUS employs FDM in advanced semiconductor devices simulations like transistors and sensors [158]. ADEPT uses FDM to simulate electronic and optical properties in semiconductor devices [107]. QUOKKA applies FDM to optimize photovoltaic device performance and efficiency [111].

Similar to FEM, the Finite Volume Method (FVM) is used by AFORHET and ASA to solve partial differential equations modeling physical phenomena such as heat diffusion and convection within solar cells [159].

All the studied software packages employ the Drift–Diffusion model to analyze charge carrier transport and device performance. This method solves the coupled Poisson and continuity equations, essential for understanding the transport dynamics of electrons and holes within the solar cell structure. SCAPS and AMPS focus on defect density and electric fields [69,160,161], while ASA and AFORS-HET emphasize material properties and layer configurations [71,159]. ASPIN3, SESAME, and GPVDM analyze carrier transport and recombination processes, providing insights into the effects of structural parameters on device performance [81,89,162,163]. SILVACO’s ATLAS and PC1D apply the model to one-dimensional simulations [164,165], while SENTAURUS extends this analysis to various geometries [166,167]. ADEPT and QUOKKA also use the drift–diffusion model to simulate carrier transport, facilitating performance simulations based on diverse material properties and configurations [168,169,170,171,172].

Many solar cell simulation tools also employ the Transfer Matrix Method (TMM) to analyze optical properties and optimize device performance. In SCAPS, the TMM models light propagation and charge carrier generation in thin-film solar cells [172]. AFORS-HET calculates optical intensity and generation rates in multilayer structures, optimizing light interaction with different layers [173]. ASPIN3 utilizes the TMM to evaluate light absorption and generation profiles, aiding in optimizing layer thickness and material properties [174]. The GPVDM uses the TMM for optical behavior analysis in multilayer designs, focusing on light propagation and electron–hole pair generation [175,176]. SILVACO’s ATLAS and SENTAURUS employ the TMM to simulate light absorption, reflection, and transmission, which is crucial for accurate device modeling and optimization [177,178,179]. QUOKKA also incorporates TMM to analyze optical properties, influencing overall device efficiency by evaluating the light interaction with various layers [180,181].

In SCAPS, the Gummel Iteration method is used to iteratively solve the Poisson equation for electric potential and the continuity equations for electron and hole densities, refining results until convergence is achieved for accurate solar cell modeling under various conditions [182,183]. Similarly, the AMPS employs the Gummel Iteration to enhance the accuracy of simulations by iteratively refining carrier concentrations and electric potential [184]. The ASA and AFORS-HET use the Gummel Iteration for efficient convergence in analyzing carrier dynamics and simulating various heterojunction configurations [185]. SILVACO’s ATLAS and SENTAURUS also utilize the Gummel method for accurate semiconductor behavior simulations, particularly in complex device structures [186,187].

The Newton–Raphson method is vital in many simulation tools for solving nonlinear equations and improving accuracy. SCAPS incorporates it within the Gummel scheme to aid in the convergence of device characteristics under different conditions [188]. The AMPS uses it to refine quasi-Fermi levels, enhancing simulation accuracy [185]. ASA, AFORS-HET, SESAME, and SENTAURUS all employ the Newton–Raphson method to handle nonlinear equations related to charge transport, recombination, and complex device modeling [71,90,189].

SCAPS also uses the Energy Balance Method to optimize energy distribution within thin-film solar cells, considering factors like light absorption and thermal losses [188]. SILVACO integrates Multi-Quantum Well structures to improve photovoltaic device efficiency [100,190,191,192].

PC1D uses Fermi–Dirac statistics for accurate simulations in highly doped regions, refining carrier distributions and band gap narrowing effects. This method is fundamental in semiconductor physics to understand how electrons (fermions) are distributed at different energy levels, especially at various temperatures, affecting conductivity and the behavior of solar cells. Implementing Fermi–Dirac statistics helps to perform more consistent and physically meaningful simulations, avoiding approximations commonly used in other models [101]. SCAPS also uses the Monte Carlo Method, which generates random events and tracks the trajectories of photons or electrons through the device. This method allows for modeling complex processes such as light absorption, luminescent emission, and the influence of geometry and type of materials on solar cell efficiency [192,193].

The S-Matrix Method is a critical component of ADEPT’s Transfer Matrix Method (TMM), ensuring numerical stability and accurate optical simulations for optimizing solar cell designs [194].

Finally, SCAPS employs the Euler Method for time-dependent simulations, which is crucial for analyzing and optimizing solar cell performance [195].

### 5.4. Cost

The cost of computational software for solar cells can vary widely depending on factors such as the specific software package, the type of license (individual, academic, commercial), the usage scope (educational, research, industrial), and any additional services or support provided. Some software packages may offer free versions or trial periods for academic or research purposes, while others may require purchasing a license or subscription for full access to all features. Pricing structures may include one-time fees, annual subscriptions, or usage-based pricing models.

Table 7 shows the cost of different software packages and licensing options.

### 5.5. Others Comparisons

QUOKKA is recognized as a “fast and easy” semiconductor simulation tool compared to Sentaurus TCAD. In performance evaluations, QUOKKA demonstrated remarkable speed, completing I–V curve computations in just 2 min on a single CPU core, while Sentaurus required 30 min on four CPU cores, making QUOKKA one to two orders of magnitude faster [86]. In terms of modeling accuracy, QUOKKA achieved V_oc_ (670 mV) and J_sc_ (37.8 mA/cm²) values nearly identical to Sentaurus, with only slight differences in the fill factor (Sentaurus: 80.0%, QUOKKA: 80.4%) and efficiency (Sentaurus: 20.3%, QUOKKA: 20.4%) [92].

In another study [110], we found minimal overall deviation (<0.2%) in the light I–V curve between Sentaurus and QUOKKA, including critical parameters such as V_oc_, J_sc_, and FF. The spatial distribution and loss breakdown analysis also showed excellent agreement between the two tools, with inaccuracies well below 1%. However, we observed that QUOKKA may not be suitable for specific scenarios, such as optimizing doping profiles or simulating intricate geometries and materials that require detailed modeling of local inhomogeneity effects [195].

For 1D cases, QUOKKA was compared with PC1D with excellent agreement of I–V curves and excess carrier densities in low and high injection [195,196]. Both Sentaurus and QUOKKA are used for solar cell simulation and optimization and offer comprehensive modeling capabilities for analyzing solar cell performance under different conditions. However, Sentaurus focuses on advanced numerical modeling and simulation techniques for semiconductor devices, while QUOKKA specializes in solving charge carrier transport in silicon devices. Also, Sentaurus offers an extensive set of models for device physics and effects in semiconductor devices, while QUOKKA includes a database of materials commonly used in thin-film solar cells.

Sentaurus TCAD is a versatile software known for its ability to predict processes with atomic-level accuracy, making it suitable for detailed simulation, even up to a sub-90 nm process. However, it comes with a higher cost, which can be a limiting factor for some users. Compared with the PC1D simulation, the Sentaurus simulation shows a low efficiency of about 0.15% abs, which is most likely because the PC1D simulation does not account for the high injection dependency of the carrier lifetime post-LID [196]. In [197], the authors show a slight deviation at high lifetimes in Sentaurus and PC1D due to lateral effects (transport of majority carriers to the point contacts at the backside), which are not accounted for in the 1D simulations with PC1D.

In [103], the authors demonstrated that PC1D accurately simulates a more significant carrier generation rate for textured surfaces than planar ones. The deviations between PC1D and Sentaurus TCAD are minimal, below 1% for planar cases and for textured depths greater than 0.1 μm. Sentaurus meticulously integrates the generation profile into a cumulative profile using a logarithmic interpolation function. However, due to the steep nature of the generation profile in the first nano- and micrometers, deviations increase at smaller depths. For textured cases, a deviation of about 10% is observed up to 10 μm in depth, with a close match at deeper depths, likely due to differences in how the two tools generate a 1D-generation rate from the 3D pyramidal geometry.

In [198], a numerical simulation of silicon-based solar cells with a degenerated SnO_2_ window layer revealed significant differences in open circuit voltage between PC1D and SCAPS. SCAPS, which is more focused on polycrystalline thin films and heterostructures, is less suitable for high doping concentrations and thick substrates, while PC1D, although suited for thick substrates, requires a more enriched database for thin film layers like fluorine-doped tin oxide.

SESAME’s performance was rigorously benchmarked against other software like SCAPS, Sentaurus, and the COMSOL Semiconductor Module, showing consistent and reliable results. For a CDS-CdTe heterojunction, the difference in the illuminated J–V curve between SESAME and Sentaurus was 0.2%, and 2% between SESAME and COMSOL. The most significant discrepancy was between SESAME and SCAPS near V_oc_, with a difference of 7%, attributed to the different interface recombination models used in SCAPS. In a 2D system with a vertical grain boundary in the CdTe layer, the difference was 1.8% between SESAME and Sentaurus and 0.7% between SESAME and COMSOL. SCAPS was not included in this analysis as it does not support 2D geometries [87].

AFORS-HET is a one-dimensional program for modeling multilayer homo- or heterojunction solar cells, offering a different approach compared to the ASA, which focuses on spatially resolved generation rate simulation in multilayer systems with sub-gap defects [199]. The ASA stands out among tools like AFORS-HET, AMPS, Sentaurus, and SCAPS for its advanced capabilities, particularly in modeling the electronic structure of hydrogenated amorphous silicon (a-Si) and hydrogenated microcrystalline silicon (μc-Si), key materials in thin-film solar cells [70]. The ASA accounts for the spatial disorder in a-Si, leading to a continuous density of states (DOS) in the energy band gap, which is crucial for accurately simulating trapping and recombination processes. It also features advanced optical modeling to optimize light management, critical for high conversion efficiencies in thin-film solar cells.

Compared to wxAMPS, SCAPS showed slight discrepancies in V_oc_, J_sc_, fill factor, and efficiency for a lead-free perovskite solar cell. SCAPS found a higher optimum absorber thickness and slightly lower maximum PCE [200]. SCAPS also provided a better description of recombination processes than the AMPS, including several tunneling mechanisms absent in the AMPS, leading to more comprehensive charge transport modeling [201], as shown in Table 8.

We identified that in the literature, there is a lack of direct comparisons to other simulation tools like ASPIN3, ADEPT, GPVDM, SC-Simul, and ASA. The provided sources focus on highlighting the strengths and features of these simulation tools.

## 6. Discussion

The analyzed programs offer robust tools for modeling various characteristics of photovoltaic devices made from different materials and technological generations, including I–V curves, quantum efficiency, carrier transport, and optical effects. Virtual simulation is essential for developing photovoltaic devices, such as first-generation solar cells and second- and third-generation thin-film solar cells, including technologies based on emerging materials (from single-junction to tandem cells). Numerical simulation is an approximated fundamental approach to assessing the feasibility of new device structures and forecasting the impact of physical changes on performance. These tools identify design issues and suggest solutions, allowing for performance evaluations before fabrication, which can save time and costs.

Simulators have enhanced the accessibility of evaluating multiple parameters—such as material type, geometric arrangement, and thickness—eliminating the need to construct every new variant physically for testing.

Most programs were designed based on the Shockley–Queisser limit model, which defines an ideal situation as a reference for actual solar cells. Any real solar cell construction is unique, with details beyond the ability of simplified schemes implemented in simulators. [202] explored how real solar cells deviate from this ideal model due to factors such as the non-absorption of some photons, thermal losses, non-radiative recombination, and internal resistances. In addition, most third-generation materials (such as perovskite, quantum dot, or plasmonic cells) can operate differently than conventional p–n junction cells, requiring a different physical approach to describe them. For example, in [15], it was analyzed that metallic nanoparticles can significantly improve the efficiency of solar cells by generating plasmons, exceeding the Shockley–Queisser limit. However, quantum corrections must be considered to obtain more accurate and realistic results when modeling this effect. Also, in [203], metallic nanoparticles were used to improve the efficiency of perovskite solar cells, observing a significant increase in efficiency, up to 40%. Here, the authors developed a theoretical model not included in any simulator software. This model was based on the coupling of plasmons with excitons in perovskite and was verified experimentally. Therefore, to effectively design and propose a new solar cell prototype, it is crucial to complement experimental data as input parameters of elected software, know the numerical methods used, and, if possible, make physical corrections to the model and compare it with experimental results.

Programs like SCAPS, AMPS, AFORS-HET, ASPIN3, GPVDM, SESAME, SILVACO, SENTAURUS, and ADEPT enable detailed simulations, assisting researchers in optimizing solar cell designs and predicting performance under varying conditions.

Tools such as ASA, AFORS-HET, SC-SIMUL, ASPIN3, GPVDM, and QUOKKA accurately model these properties, addressing critical aspects like band diagrams, quantum efficiency, spectral response, light scattering, and electrical transport phenomena, including current–voltage (I–V) curves. These factors are vital for optimizing cell performance, as carrier recombination and series and shunt resistances significantly affect energy conversion efficiency [204].

However, the simulation of multi-junction solar cells, crucial for advanced high-performance devices, is not supported by some programs like ASA, AFORS-HET, SC-SIMUL, ASPIN3, GPVDM, and QUOKKA, limiting their applicability in the development of multi-layered cells [78,205]. Tools such as SCAPS, which include noise modeling, are helpful for analyzing device behavior under different stress conditions [206]. Additionally, the simulation of degradation and thermal effects, available only in a few programs like GPVDM and SESAME, allows for predicting the device’s longevity and stability over time, which is crucial for commercial implementation [207]. Therefore, the choice of software directly influences the ability to predict and optimize the actual performance of thin-film solar cells.

The simulation of these cells requires numerical precision to capture complex phenomena such as charge transport and light interaction with the device structure. Among numerical methods, the Finite Difference Method (FDM), known for its simplicity in solving differential equations, is not implemented in some programs like AFORS-HET and SC-SIMUL, limiting their ability to model specific transport and recombination phenomena [208]. On the other hand, the Finite Volume Method (FVM), known for its accuracy in conserving physical quantities, is only present in ASA and AFORS-HET, restricting its use in other programs that could benefit from this method to solve charge flow problems [209]. The Euler Method, used in SCAPS, provides a simple yet effective approximation in some instances of carrier transport [208].

The Drift–Diffusion Method, essential for modeling carrier movement in solar cells, is absent in SC-SIMUL, limiting its ability to accurately simulate charge dynamics under electric fields [210]. Furthermore, AMPS, ASA, and SC-SIMUL tools lack the Transfer Matrix Method (TMM), crucial for modeling light interaction in thin layers. This trait affects the simulation of optical efficiency and light trapping [211,212]. Only PC1D contains the S-Matrix method, restricting the analysis of optical wave scattering [213].

Iterative methods, such as Gummel Iteration and Newton–Raphson, are essential for solving nonlinear systems. However, the lack of Gummel Iteration in programs like SC-SIMUL and others limits the rapid convergence of solutions to complex problems [212]. Fermi–Dirac statistics, vital for modeling carrier behavior under high-density conditions, are only available in PC1D, restricting detailed analysis in other programs. Multi-Quantum Wells (MQW) modeling, important for advanced cells, is only available in SILVACO, reflecting a limitation in modeling complex thin-film structures in other tools [214].

These differences in numerical methods significantly impact the accuracy and scope of simulations, affecting the development and optimization of thin-film solar cells in terms of performance and stability. When comparing programs used to simulate these cells, it is essential to consider three key factors: accuracy, costs, and processing speed. Programs like SILVACO and SENTAURUS, offering advanced modeling of multi-layer structures, and methods like MQW and TMM, provide highly accurate simulations in light interaction and charge transport [214]. However, these levels of detail often come with longer processing times and require commercial licenses, which may be prohibitive for some researchers and small companies [215].

Regarding usability, tools like SCAPS, PC1D, and QUOKKA are preferred due to their intuitive interfaces and minimal setup, making them suitable for quick simulations of thin-film and silicon-based photovoltaic devices. On the other hand, SILVACO and SENTAURUS TCAD offer advanced performance for highly detailed 3D simulations, such as those required for multi-junction or nanostructured solar cells but necessitate more significant expertise and computational power. Simulators like SC-SIMUL and AFORS-HET are specialized for complex heterojunction and multilayer devices, providing essential tools for studies focused on interface effects. Meanwhile, the GPVDM balances ease of use and functionality, offering versatility in simulating organic and inorganic solar cells with 2D and 3D capabilities.

In terms of costs, open-source or low-cost software like SCAPS and AFORS-HET are more accessible. Still, their accuracy may be limited by the absence of advanced features such as degradation modeling or the simulation of thermal stress and multiscale effects [216]. On the other hand, commercial programs like SILVACO and SENTAURUS, though more expensive, offer technical support and regular updates, which may justify their investment in industrial projects or advanced research.

Regarding speed, tools like SC-SIMUL and the ASA tend to be faster due to their lower complexity, making them attractive options for quick simulations or preliminary studies. However, this speed may sacrifice depth in modeling more complex devices. In contrast, programs like SILVACO or the GPVDM, which include methods such as Newton+-Raphson or Gummel Iteration, tend to be slower but offer more detailed and robust results, especially in cells with advanced geometries [217].

One of the critical challenges in simulating thin-film solar cells is translating the simulated results into the real world. Numerical models require assumptions and simplifications that do not always fully represent the complex behaviors of solar cells under real conditions, such as temperature degradation or solar light variability. Tools like SILVACO and SENTAURUS, which allow for advanced simulations with multiple variables, offer results closer to reality but at a high cost and time [218]. This trait poses a dilemma for researchers seeking a balance between accuracy and economic viability. SCAPS, AMPS, SILVACO, and SENTAURUS TCAD present significant challenges, mainly when modeling complex phenomena like plasmonic photovoltaic effects in perovskite cells. Meanwhile, these simulators perform well for standard materials.

In conclusion, selecting the appropriate simulation software for solar cells requires balancing accuracy, cost, speed, and usability. Tools like SILVACO and SENTAURUS offer advanced and detailed simulations, which are ideal for complex research but expensive. At the same time, programs such as SCAPS and AFORS-HET provide more accessible options, though with functional limitations. Understanding the capabilities and constraints of each simulator enables researchers to optimize the design and development of photovoltaic devices more efficiently.

## 7. Conclusions

In conclusion, simulation programs like SCAPS, AMPS, AFORS-HET, ASPIN3, GPVDM, SESAME, SILVACO, SENTAURUS, and ADEPT are essential for modeling and analyzing solar cell performance. They offer detailed simulations of types of materials and technologies, band diagrams, capacitance, I–V curves, quantum efficiency, carrier transport, and more, facilitating the optimization of solar cell designs and performance predictions. Despite these advances, challenges persist in modeling complex phenomena such as degradation, multi-junction effects, and stress impacts. Advanced numerical methods like the FEM and FDM, along with the Drift–Diffusion model, are critical, but further refinements are needed to tackle stress effects and doping profile optimization. Cost and accessibility are significant factors, with some programs available for free while others involve substantial investment. These tools varying in computational efficiency and accuracy highlight the need to choose the right software based on research goals and budgets. Continued development and accessibility of these tools will be crucial for advancing photovoltaic technology.

## Figures and Tables

**Table 1:** Classification of materials for solar cells.

First Generation Uses Inorganic Semiconductor Materials in Bulk	Second Generation Uses Thin-Film Inorganic Semiconductor Materials	Third Generation Uses Organic, Inorganic, and Hybrid Semiconductor Materials	Third Generation/Emerging Include Technologies for the New Generations
Based on Crystalline Silicon (c-Si) [18]	Based on Thin-Film Silicon or Amorphous Silicon (a-Si) [19]	Perovskite Solar Cells [20]	Nanostructured Solar Cells (Nanocrystals, Nanowire, Nanotubes, Nanorods, Nanofiber, etc.) [21,22]
Based on Polycrystalline Silicon [18]	Based on binary compounds: IV–IV, III–V, II–VI and IV–VI (GaAs, CdTe, etc.) [23]	Organic Photovoltaics (OPV): Carbon-Based Materials, Fullerenes, Polymers and Small Molecules [17,21]	Plasmonic Solar Cells [15]
Based on Heterojunction with Intrinsic Thin layer (HJT) [13]	Based on Kesterite: Copper, Zinc, Tin, Sulfide or Selenide or Sulfoselenide (CZTS, CZTSe, CZTSSe) [24]	Dye-Sensitized Solar Cells (DSSC) [25]	Flexible, Ultra-Thin, Ultra-Light, 3D-Printable Solar Cells [26]
Based on Gallium Arsenide (GaAs) [27]	Based on Titanium Oxide (TiO_2_) [27]	Quantum Dot Solar Cells [17]	Transparent and Semi-Transparent Solar Cells [23,26]
	Based on Gallium Arsenide Selenide (GaAsSe) [28]	Tandem Solar Cells [29]	Photonic Crystal Solar Cells [30]
	Based on Chalcogenides: Sulfides, Selenides, Tellurides (CdTe, CuS, SnS, MoS, etc.) [31]	Multi-Junction Solar Cells [23,29]	Black Silicon Solar Cells [32]
	Based on Chalcopyrite: Copper, Indium, Gallium, Selenide (CIS, CIGS) [33]	Hybrid Solar Cells [11]	Solar Cells based on Graphene, Graphene Oxide (GO), reduced Graphene (rGO), Graphite, and Nano-Graphite [34]
			Hot Carrier Solar Cells [35]
			Luminescent Solar Concentrators [36]

**Table 2:** Strengths, weaknesses, and uses of different types of modeling employed in thin-film solar cell research.

Type of Modelling	Strengths	Weaknesses	Uses in Research
Band Diagram Modeling [38]	Allows visualization of band alignment and potential barriers.	Difficult to apply in complex materials, multiple layers, or heterostructures.	Device design and analysis of carrier transport efficiency.
Quantum Efficiency (QE) Modeling [39]	Analyzes the fraction of photons that generate useful charge carriers.	Does not account for other effects like recombination or resistive losses.	Study of spectral response and photon-to-current conversion.
Spectral Response Modeling [40]	Allows measurement of efficiency at different wavelengths.	Does not account for thermal losses or recombination effects.	Evaluation of spectral efficiency under various solar light conditions.
Optical Modeling [41]	Simulates light absorption and reflection within the cell structure.	Limited in long-term simulations or extreme operating conditions.	Optimization of light absorption to maximize quantum efficiency.
Light Trapping and Scattering Modeling [41]	Optimizes light capture in thin-film cells.	Complex to implement in advanced geometries.	Maximization of light absorption in thin-film structures.
I–V Modeling [41]	Provides information on efficiency, short-circuit current, and open-circuit voltage.	Insufficient for modeling dynamic or transient effects.	Characterization of overall device efficiency under different light conditions.
Electrical Modeling [41]	Studies the general electrical behavior of the device under different conditions.	Does not capture all optical or thermal phenomena.	Overall evaluation of electrical efficiency and performance under operating conditions.
Carrier Transport Modeling [42]	Allows detailed analysis of electron and hole movement within the cell.	Difficult to implement in devices with complex geometries or materials.	Simulation of charge transport to improve carrier mobility.
Recombination Mechanism Modeling [40]	Analyzes the rates and mechanisms of recombination within the device.	Difficult to model accurately in non-conventional materials.	Study of recombination to minimize losses in cell efficiency.
Series and Shunt Resistance Modeling [43]	Provides information on resistive losses within the device.	Cannot capture other non-resistive loss mechanisms.	Optimization of series and shunt resistances to improve conversion efficiency.
Material Properties Modeling [44]	Allows analysis of the impact of material properties on overall performance.	Requires precise data for the materials used.	Simulation of new materials or material combinations to improve efficiency.
Capacitance Modeling [45]	Useful for studying junction capacitance and behavior in response to frequencies.	Limited to specific operating conditions.	Analysis of capacitance as a function of frequency to characterize junction quality.
ETL and HTL Modeling [45]	Enables detailed analysis of electron and hole transport through selective layers.	Difficult to model interfaces and defects between layers accurately.	Optimization of ETL and HTL materials for improving charge carrier selectivity, minimizing recombination, and enhancing overall device efficiency.
Doping and Defect Modeling [46]	Evaluates the effect of doping and defects on cell performance.	It requires precise data and is difficult to validate experimentally.	Study of the impact of doping levels and defects on efficiency and device lifetime.
Interface Modeling [42]	Evaluates behavior at interfaces between different material layers.	Complex to simulate multiple interfaces.	Improvement in efficiency and reduction in recombination losses at interfaces.
Multi-Junction Modeling [45]	Studies the behavior of multi-junction devices to optimize efficiency.	Complexity in simulating multiple junctions.	Research of high-efficiency multi-junction solar cells.
Thermal Modeling [43]	Studies the effect of heat on device performance.	Difficult to integrate with optical or electrical models in complex simulations.	Simulation of behavior under extreme or fluctuating thermal conditions.
Transient Response Modeling [40]	Analyzes device behavior under rapid changes in illumination conditions.	Does not fully capture long-term effects.	Study of device response under fluctuating light conditions.
Photocurrent and Photovoltage Modeling [41]	Evaluates current and voltage generation under different lighting conditions.	Does not fully model long-term effects or degradation.	Optimization of the balance between photocurrent and photovoltage.
Lifetime and Degradation [44] Modeling	Evaluates long-term durability and efficiency.	Requires precise and long-term data, making implementation challenging.	Study of lifetime and degradation in efficiency over time.
Multiscale Modeling [42]	Integrates phenomena across different scales into a single simulation.	High computational load and difficult to validate experimentally.	Analysis of effects occurring at different spatial and temporal scales within the device.
Stress Effects Modeling [43]	Studies the impact of mechanical stresses on device structure.	Cannot capture all microstructural effects.	Analysis of structural integrity and mechanical durability under variable operating conditions.
Noise Modeling [44]	Analyzes the impact of electrical noise on device performance.	Relevant primarily in very high-efficiency devices.	Study of noise in the device to reduce interference.

**Table 3:** Strengths, weaknesses, and uses of different types of numerical methods employed in thin-film solar cell research.

Type of Modelling	Strengths	Weaknesses	Uses in Research
Finite Element Method (FEM) [52]	High accuracy for complex geometries and material properties; flexible meshing.	Computationally expensive, especially for large-scale problems.	Used in modeling stress, strain, and electric fields.
Finite Difference Method (FDM) [53]	Simple to implement; suitable for problems with regular geometries and grid structures.	Difficult to apply to complex geometries; limited accuracy in regions with sharp changes.	Solving time-dependent diffusion equations in drift–diffusion models of thin-film solar cells.
Finite Volume Method (FVM) [54]	Conserves fluxes across control volumes; suitable for problems involving conservation laws.	Requires structured grid; can be less accurate near boundaries.	Modeling the electrostatic potential and charge transport in thin-film solar cells.
Euler Method [55]	Easy to implement and fast for simple problems.	Low accuracy; highly dependent on time step size; unstable for stiff problems.	Basic drift–diffusion simulations in solar cells when high precision is not required.
Drift–Diffusion Modeling [39]	Provides a detailed representation of charge carrier transport under electric fields.	Computationally demanding; requires precise knowledge of material parameters.	Carrier transport analysis and efficiency prediction in thin-film solar cells.
Transfer Matrix Method (TMM) [56]	Efficient for calculating optical properties in multi-layered thin-film structures.	Only applicable to planar, periodic structures; assumes perfect interfaces.	Optical absorption and reflectivity analysis in thin-film solar cells.
S-Matrix Method [57]	Accurate for analyzing scattering properties of multi-layered media; stable numerical method.	Requires complex computations; limited applicability to highly disordered structures.	Optical analysis of reflection and transmission in multi-layered thin films.
Gummel’s Method [58]	Iterative method suited for solving Poisson’s equation in semiconductor devices.	Convergence can be slow for heavily doped regions; limited to low-injection conditions.	Used in solving semiconductor device equations in thin-film solar cells.
Newton–Raphson Method [55]	Fast convergence for nonlinear problems; useful for refining solutions in iterative processes.	May not converge if initial guess is poor; computationally expensive for large systems.	Applied to solving nonlinear drift–diffusion equations in thin-film solar cells.
Fermi–Dirac Statistics [59]	Essential for modeling charge carriers in semiconductors, especially at quantum scale.	Difficult to apply without proper understanding of quantum mechanics; complex to solve numerically.	Carrier distribution modeling in highly doped or quantum-confined thin-film solar cells.
Multi-Quantum Well structures (MQW) [60]	Provides enhanced optical absorption and carrier confinement in thin layers.	Requires complex fabrication techniques and precise quantum mechanical modeling.	Enhancing absorption in thin-film solar cells through quantum well engineering.

**Table 4:** Photovoltaic cells and materials in each software for thin-film solar cells.

Software	1st Generation Materials	2nd Generation Materials	3rd Generation Materials
SCAPS	Si, GaAs	CdTe, CIS, CIGS, CZTS	Kesterite, Perovzkite
AMPS	Si, GaAs	CdTe, CIGS, CZTS	CZTS
ASA	Si, GaAs	CdTe, CIGS	Multi-layer heterojunction
AFORS-HET	Si, GaAs	CdTe, CIGS, a-Si	a-Si
SC-SIMUL	Si, GaAs	CdTe, CIGS	a-Si
ASPIN3	Si, GaAs	CdTe, CIGS	LEDs and lasers
GPVDM	Si, GaAs	CdTe, CIGS	Perovskite, Organic
SESAME	Si, GaAs	CdTe, CIGS, Perovskite	Perovskite
SILVACO	Si, GaAs	a-Si, CdTe, CIGS	Multi-materials
PC1D	Si	a-Si	Ge
SENTAURUS	Si, GaAs	CdTe, CIGS	Multi-materials
ADEPT	Si, GaAs	a-Si, CdTe, CIS	Multi-junction
QUOKKA	Si	a-Si, CdTe, CIGS	Quasi-neutral Si

**Table 5:** Types of modeling used in the simulation of thin-film solar cells.

Modeling	SCAPS	AMPS	ASA	AFORS-HET	SC-SIMUL	ASPIN3	GPVDM	SESAME	SILVACO	PC1D	SENTAURUS	ADEPT	QUOKKA
1. Electronic and Optical Properties Modeling													
Band Diagram Modeling	x	x	x	x	x	x	x	x	x	x	x	x	x
Quantum Efficiency Modeling	x	x	x	x	x	x	x	x	x	x	x	x	x
Spectral Response Modeling	x	x	x	x	x	x	x	x	x	x	x	x	x
Optical Modeling	x	x	x	x	x	x	x	x	x	x	x	x	x
Light Trapping and Scattering Modeling	x	x	x	x	x	x	x	x	x	x	x	x	x
2. Electrical and Transport Phenomena Modeling													
Current–Voltage (I–V) Modeling	x	x	x	x	x	x	x	x	x	x	x	x	x
Electrical Modeling	x	x	x	x	x	x	x	x	x	x	x	x	x
Carrier Transport Modeling	x	x	x	x	x	x	x	x	x	x	x	x	x
Recombination Mechanism Modeling	x	x	x	x	x	x	x	x	x	x	x	x	x
Series and Shunt Resistance Modeling	x	x	x	x	x	x	x	x	x	x	x	x	x
Material Properties Modeling	x	x	x	x	x	x	x	x	x	x	x	x	x
Capacitance Modeling	x	x	x	x	x	x	x	x	x	x	x	x	x
3. Device Structure and Interface Modeling													
Absorber Layer Modeling	x	x	x	x	x	x	x	x	x	x	x	x	x
Doping and Defect Modeling	x	x	x	x	x	x	x	x	x	x	x	x	x
Interface Modeling	x	x	x	x	x	x	x	x	x	x	x	x	x
Multi-Junction Modeling	x	x		x				x	x	x	x	x	
Lifetime Modeling	x	x		x		x	x	x	x		x	x	
ETL and HTL Modeling	x			x				x	x		x	x	
4. Thermal and Transient Response Modeling													
Thermal Modeling	x	x	x	x	x	x	x	x	x	x	x	x	x
Transient Response Modeling	x	x	x	x	x	x	x	x	x	x	x	x	x
5. Performance Metric Modeling													
Photocurrent and Photovoltage Modeling	x	x	x	x	x	x	x	x	x	x	x	x	x
Degradation Modeling							x						
6. Multiscale and Noise Modelling													
Multiscale Modeling								x				x	
Stress Effects	x	x	x			x	x		x	x		x	x
Noise Modeling	x												

**Table 6:** Numerical methods used in the simulation of thin-film solar cells.

Modeling	SCAPS	AMPS	ASA	AFORS-HET	SC-SIMUL	ASPIN3	GPVDM	SESAME	SILVACO	PC1D	SENTAURUS	ADEPT	QUOKKA
1. Numerical Methods for Differential Equations													
FEM	x	x	x	x	x	x	x	x	x	x	x	x	x
Finite Difference Method	x	x	x			x	x	x	x	x	x	x	x
FVM			x	x									
Euler Method	x												
Drift–Diffusion Method	x	x	x	x		x	x	x	x	x	x	x	x
2. Matrix and Interactive Methods													
Transfer Matrix Method	x			x		x	x		x		x		x
S-Matrix Method										x			
Gummel Iteration	x	x	x	x					x		x		
Newton Rapson	x	x	x	x				x			x		
3. Statistical and Quantum Mechanic Methods													
Fermi–Dirac Statistics										x			
Monte Carlo Method	x												
4. Advanced Structures and Materials Mode													
MQW									x				

**Table 7:** Cost of different simulators for thin-film solar cells.

Software	Source	Availability
One-dimensions		
SCAPS	http://scaps.elis.ugent.be/ (accessed on 4 October 2024).	Free and open source
AMPS/wxAMPS	https://github.com/wxAMPS (accessed on 4 October 2024).	USD 0 per month for basics for individuals and organizations USD 3.67 per user/month for the first 12 months for advanced collaboration for individuals and organizations USD 19.35 per user/month for the first 12 months for security, compliance, and flexible deployment
ASA	https://asa.ewi.tudelft.nl/ (accessed on 4 October 2024).	Command-line-driven software
AFORS-HET	https://www.helmholtz-berlin.de/forschung/oe/se/silizium-photovoltaik/projekte/asicsi/afors-het/download/index_en.html (accessed on 4 October 2024).	Free and open source
SC-SIMUL	http://www.greco.uni-oldenburg.de/download.html (accessed on 4 October 2024).	Free and open source
Two-dimensions		
ASPIN3	http://lpvo.fe.uni-lj.si/en/software/aspin3/ (accessed on 4 October 2024).	Demo version
GPVDM	https://www.oghma-nano.com/download.php (accessed on 4 October 2024).	Free source
SESAME	https://pages.nist.gov/sesame/ (accessed on 4 October 2024).	USD 0 per month for basics for individuals and organizations USD 3.67 per user/month for the first 12 months for advanced collaboration for individuals and organizations USD 19.25 per user/month for the first 12 months for security, compliance, and flexible deployment
Three-dimensions		
SILVACO	https://dynamic.silvaco.com/dynamicweb/silen/ (accessed on 4 October 2024).	There are various licensing models, such as perpetual licenses, subscription-based licenses, or academic licenses. The cost can also depend on the size and type of organization (e.g., educational institution, research organization, commercial company).
PC1D/PC3D	https://www.engineering.unsw.edu.au/energy-engineering/research/software-data-links/pc1d-software-for-modelling-a-solar-cell (accessed on 4 October 2024).	It is freely available for academic and educational purposes. Commercial users or organizations may need to purchase a license, which can vary depending on the organization’s size, intended usage, and specific licensing requirements.
SENTAURUS	www.synopsys.com/support/training/dfm/basic-training-on-tcad-sentaurus-tools.html (accessed on 4 October 2024).	Universities and research institutions may have access to academic licenses or discounted rates for academic and research purposes. For commercial usage, the cost involves purchasing licenses or subscriptions based on the organization’s size, intended usage, and specific requirements. The pricing structure may include upfront license fees, annual maintenance fees, and additional technical support and updates fees.
ADEPT	https://nanohub.org/tools/adeptnpt (accessed on 4 October 2024).	The costs vary depending on the license type (individual, institutional, commercial), the scope of usage (academic, research, commercial), and any additional services or support provided.
QUOKKA	https://www.quokka3.com/purchase/license-options.html (accessed on 4 October 2024).	It is freely available for academic and educational purposes. Licensed for commercial use

**Table 8:** Comparison of SCAPS and wxAMPS [201].

	V_oc_ (V)			J_sc_ (mA/cm^2^)			FF (%)			FPE (%)		
Cell Configuration	SCAPS	AMPS	Exp.	SCAPS	AMPS	Exp.	SCAPS	AMPS	Exp.	SCAPS	AMPS	Exp.
FTO/TiO_2_/CH_3_NH_3_PbI_3_/Spiro/Au	1.12	1.27	1.02	24.32	21.58	21.20	81.86	79.00	77.60	22.35	20.00	18.70
FTO/TiO_2_/CH_3_NH_3_PbI_3_/Au	0.85	0.90	0.82	20.19	22.95	18.10	82.88	81.49	78.20	1770	17.01	12.60
FTO/TiO_2_/CH_3_NH_3_PbI_3_/CuSCN/Au	1.21	1.24	1.10	20.62	23.19	19.70	79.79	77.72	75.00	20.00	22.38	18.40
FTO/TiO_2_/CH_3_NH_3_PbI_3_/CiI/Au	1.01	1.07	0.95	21.31	23.08	19.80	80.77	78.64	76.00	17.54	19.60	15.50
FTO/TiO_2_/CH_3_NH_3_PbI_3_/NiO/Au	1.01	1.13	0.93	20.23	22.00	18.90	81.46	79.38	77.00	17.28	19.89	16.20
FTO/ZnO/CH_3_NH_3_PbI_3_/NiO/Au	0.99	1.04		25.62	26.02		80.03	79.45		21.87	20.67	
FTO/SnO_2_/CH_3_NH_3_PbI_3_/NiO/Au	0.99	1.02		25.73	25.87		75.45	74.83		19.36	18.69	
